# National Variation in Black Immigrant Preterm Births and the Role of County-Level Social Factors

**DOI:** 10.1007/s40615-024-02198-4

**Published:** 2024-10-08

**Authors:** Ozi Amuzie, Joshua Radack, Nancy Yang, Alejandra Barreto, Daria Murosko, Sara C. Handley, Scott A. Lorch, Heather H. Burris, Diana Montoya-Williams

**Affiliations:** 1 Division of Neonatology, Children’s Hospital of Philadelphia, 2716 South Street, Philadelphia, PA 19146, USA; 2 School of Medicine, University of California, San Francisco, San Francisco, CA, USA; 3 Department of Pediatrics, Perelman School of Medicine at University of Pennsylvania, Philadelphia, PA, USA; 4 Children’s Hospital of Philadelphia PolicyLab, Philadelphia, PA, USA

**Keywords:** Preterm birth, Prematurity, Immigrant paradox, Birth outcomes, Racial disparity, Health equity

## Abstract

Preterm birth rates among Black individuals continue to be inequitably high in the USA. Black immigrants appear to have a preterm birth advantage over US-born counterparts. This national cross-sectional study of singleton non-Hispanic Black individuals in the USA from 2011 to 2018 aimed to investigate if the Black immigrant preterm birth advantage varied geographically and how this advantage associated with county-level social drivers of health. Generalized linear mixed models explored the odds of preterm birth (< 37 weeks) by birthing person’s nativity, defined as US- versus foreign-born. In county-level analyses, five measures were explored as possible sources of structural risk for or resilience against preterm birth: percent of residents in poverty, percent uninsured, percent with more than a high school education, percent foreign-born, and racial polarization. County-level immigrant advantage among foreign-born compared to US-born Black individuals was defined by a disparity rate ratio (RR); RR < 1 indicated a county-level immigrant preterm birth advantage. Linear regression models at the level of counties quantified associations between county-level factors and disparity RRs. Among 4,072,326 non-Hispanic Black birthing individuals, immigrants had 24% lower adjusted odds of preterm birth compared to US-born Black individuals (aOR 0.77, 95% CI 0.76–0.78). In county-level analyses, the immigrant advantage varied across counties; disparity RRs ranged from 0.13 to 2.82. County-level lack of health insurance and education greater than high school were both associated with immigrant preterm birth advantage. Future research should explore policies within counties that impact risk of preterm birth for both US-born and immigrant Black individuals.

## Introduction

In the USA, Black birthing people have substantially higher rates of preterm birth (PTB) compared to any other racial or ethnic group. In 2021, PTB rates among non-Hispanic Black people were 55% higher than non-Hispanic White people [1]. Prior studies have demonstrated that foreign-born Black people in the US have lower rates of poor birth outcomes, including PTB, compared to their US-born Black counterparts [2–4]. This immigrant advantage is a global phenomenon; despite having a higher likelihood of socioeconomic disparities and structural barriers to healthcare in the receiving country, immigrants within a racial or ethnic subgroup have similar or better health outcomes than their native-born counterparts [5].

The immigrant paradox is likely related to a combination of individual factors (e.g., protective health behaviors) and neighborhood-level factors (e.g., immigrant community cohesion) that together confer individual and structural resilience to adverse outcomes like PTB [6]. Structural resilience has been described as the converse of structural discrimination; factors at the community level such as social networks, neighborhood dynamics, and local policies, which may promote health [7]. With respect to immigrant health, living in co-ethnic immigrant enclaves (areas with high concentrations of immigrants who share an ethnicity) may represent a form of structural resilience. Relevant to birth outcomes, there are data suggesting that residence in a co-ethnic enclave is associated with higher birthweights among immigrants of various backgrounds [8–10]. However, immigrant birth outcomes are also negatively affected by structural forms of discrimination that exist where a pregnant person lives, such as restrictive state policies regarding insurance eligibility [11, 12].

Despite the epidemiologic advantages, Black immigrant still experience higher rates of PTB compared to non-Black immigrants [13, 14]. Structural discrimination is critical to the study of birth outcomes among Black individuals, as it is a modifiable root cause of racial health disparities [15, 16]. Structural racism is one type of structural discrimination and refers to the patterns, practices, and policies which systematically distribute resources away from Black people and other marginalized races and increase risks for poor health [17]. Previous literature has documented a relationship between PTB among Black birthing people and area-level risk factors thought to represent structural discrimination such as racial segregation and neighborhood poverty [18–20]. However, the contribution of structural discrimination to immigrant birth outcomes is less clear [21, 22]. Furthermore, it is critical to examine how structural forms of both advantage and disadvantage differentially manifests across geographic contexts [17].

Preterm birth is intricately related to a birthing parent’s health; it is both an outcome of prenatal morbidity such as related to hypertensive disorders and also directly increases risk of morbidity in subsequent pregnancies [23]. Given the ongoing birthing parent morbidity crisis and its disproportionate impact on Black communities [24], understanding what may be associated with or protective against preterm birth in Black birthing people at a population level may inform policy solutions to mitigate this public health crisis. Thus, in this study, we examined the Black immigrant PTB advantage and its structural drivers in a multiyear national dataset. Although the Black immigrant birth outcomes paradox has been documented using state-level datasets [14, 25–29], few have used national datasets [30] in a multilevel way that accounts for both individual and structural-level drivers of disparities. Secondarily, we aimed to explore how the Black immigrant paradox with respect to PTB varies across the country and how county-level characteristics may function as markers of either area-level structural risk or resilience for Black immigrant communities. We hypothesized that the Black immigrant PTB advantage would be significantly associated with county-level markers of both structural risk and resilience.

## Methods

### Study Population

This was a cross-sectional observational study of restricted-use national vital statistics data for singleton births to non-Hispanic Black birthing individuals in the USA from 2011 to 2018. Birth certificates were revised in 2003, but the uptake of the revision varied across the country over time [31]. Because availability of nativity data varied between the unrevised and revised birth certificates, only data from states that had adopted the 2003 revision were included. This represented 91.1% of all births in the USA during the study period. Birth records were excluded if missing nativity or birthing person’s county of residence or if the gestational age was < 22 or > 45 weeks.

### Study Variables

Our study was foundationally based on the social ecological model of health, which posits that health outcomes and disparities are impacted not only by individual factors but also by the structures and systems in individuals’ environment [32]. Racial birth outcome disparities thus reflect how racially minoritized pregnant people are discriminated against in individual and structural ways [33]. To select specific variables for this study, we drew from Kramer and Hogue’s framework for racial disparities in PTB, which outlines how phenotypic race may be associated with increased risk of PTB through pathways related to different forms of racism [34]. We also used the vulnerability and resilience conceptual model for migrant health developed by the International Organization for Migration [35] to help us consider individual and structural risk factors for PTB among immigrants as well as structural resilience factors or factors that might mitigate the risk of PTB for immigrants.

Given our aims, this study consisted of (1) individual-level analyses estimating foreign-born Black people’s risk of preterm birth based on individual and county-level variables and (2) county-level analyses estimating county-level nativity disparities associated with county-level variables. For individual-level analyses, the primary exposure was birthing parent nativity, defined as US- versus foreign-born. The primary outcome of interest was PTB (birth < 37 weeks’ gestation). Individual-level covariates were selected based on existing associations with PTB and included age, pre-pregnancy body mass index, smoking during pregnancy, prenatal care adequacy, insurance, education, and marital status [36–41]. Although these may be on the causal pathway between nativity and differential risk of preterm birth, we aimed to explore the marginal effect outside these known associations. We also controlled for congenital anomalies, rather than excluded births with these, to preserve sample sizes. Missing values for any of these covariates were included as missing indicators.

Using the aforementioned conceptual frameworks [34, 35], we also selected several county-level factors that might be impacting the risk of preterm birth for individuals living within that county. In order to avoid solely deficit-based models [42], racial segregation, presence of an immigrant enclave, and both area-level socioeconomic disadvantage and advantage. Counties were chosen as the unit of analysis for area-level factors for three reasons. First, this optimized cell sizes to statistically evaluate a relatively rare outcome (i.e., PTB) among a small population of birthing people (i.e., Black immigrants). Second, the impact of structural measures on health outcomes at the county-level is well established in the literature [43, 44]. Finally, counties represent an actionable level for policy implementation given many public health departments are administered by counties [45].

**Fig. 1:**
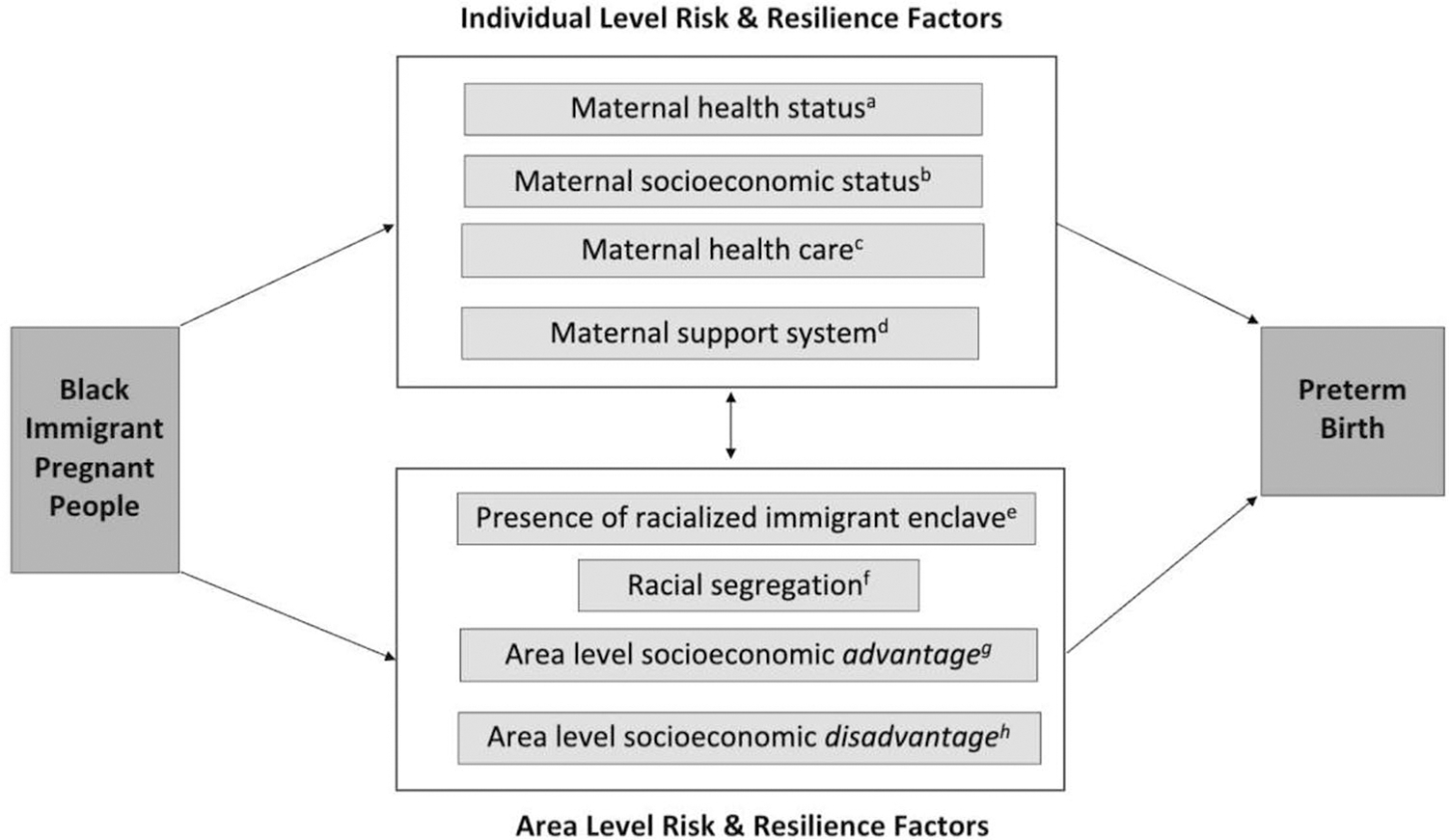
Constructs associated with preterm birth risk among Black immigrant birthing people selected for this study. Legend: This study relied on two existing conceptual frameworks to select variables that serve as proxies for a variety of constructs associated with preterm birth risk among Black immigrant birthing people. Variables that reflect both individual-level factors and the geographic area in which a birthing person lives (county) were included in this study. ^a^Birthing parent age, pre-pregnancy BMI, smoking status; ^b^birthing parent insurance, education; ^c^prenatal care adequacy; ^d^marital status; ^e^percent of Black foreign-born individuals in county of residence; ^f^county-level racial Index of Concentration of the Extremes (ICE_race_); ^g^percent of individuals in county with more than a high school education; ^h^percent of uninsured individuals in county

Five county-level exposure variables were obtained from the 2013–2017 5-year American Community Survey and were standard deviation normalized. Area-level socioeconomic disadvantage in this study was defined as percent of the county’s population: (1) living below the poverty line and (2) without health insurance. Area-level socioeconomic advantage was defined as percent of the county’s population having more than a high school education. We used percent of county residents who were Black and foreign-born as a proxy for the likelihood of the presence of a county-level racialized immigrant enclave, a novel approach. Racial segregation was quantified by the Index of Concentration of Extremes (ICE), one of the most frequently used measures to examine segregation as a driver of health disparities [46, 47]. ICE measures polarization in an area by considering both advantage and disadvantage in its calculation [48]. ICE ranges from − 1 (most disadvantaged) to 1 (most privileged). In this study, we used I CE_race_, with − 1 representing a county where 100% of residents self-reported being Black and 1 representing a county where 100% of residents self-reported as White. Further information about ICE is in Online Resource 1. Figure 1 summarizes both the individual-level variables we adjusted for as well as the county-level exposure variables selected for this study and the constructs for which they serve as a proxy.

### Analyses

We used standardized mean differences (SMDs) to compare foreign-born and US-born people, since very small differences can be statistically significant (*P* < 0.05) when using chi-square tests in large cohorts. An SMD > 0.1 was considered significant [49].

In the first set of individual-level analyses, we examined the association of individual foreign-born nativity with PTB among Black birthing people to confirm the immigrant advantage in this national cohort. We performed a series of multilevel generalized linear mixed models to estimate the odds of preterm birth for foreign-born Black people compared to US-born Black people after adjusting for individual characteristics, fixed effects related to birth state and year, and random effects that allowed for clustering of births by county. We first fit unadjusted models (model 1); then adjusted for maternal characteristics, congenital anomalies, birth state, and birth year along with a random effect for county (model 2); and made additional adjustment for each county-level exposure (models 3–7).

In the county-level analyses to examine US geographic variability in the county-level Black foreign-born PTB advantage, we analyzed counties with ≥ 100 births among Black individuals and ≥ 25 births to foreign-born Black individuals to increase the chances of detecting PTBs and minimize the bias introduced by outlier counties with few Black births. We calculated county-level disparity rate ratios to assess disparities in PTB between foreign and US-born individuals in each county, as others have done to assess relative inequities between two groups [50–52]. The PTB rate ratio consisted of the foreign-born PTB rate in the numerator and the US-born PTB rate in the denominator. A rate ratio < 1 indicates lower PTB risk for foreign-born Black people relative to US-born people (a relative county-level immigrant PTB advantage). This could mean either lower PTB rates among foreign-born or higher PTB rates among US-born individuals. Conversely, a ratio > 1 indicates higher PTB risk among foreign-born people relative to US-born people at the county level (a relative immigrant PTB disadvantage). Online Resource 1 further details on this county-level disparity rate ratio.

We fit linear regression models to explore the unadjusted relationship between each county-level exposure variable and county PTB disparity rate ratio. The county PTB rate ratio was log transformed, based on a Box Cox analysis, to obtain normally distributed residuals [53]. We excluded counties with zero PTBs to either foreign-born or US-born Black individuals to allow for the calculation of the rate ratio. In these models, each county-level factor was first introduced separately in unadjusted models. A final adjusted county-level model was built that included all five county-level variables and their combined effect on the county PTB rate ratio. To confirm findings from these models, we conducted sensitivity analyses using Bayesian estimations of county PTB disparity rate ratios. This allowed the inclusion of counties previously excluded for having zero PTBs and adjusted for the bias introduced by counties with very few PTBs (Online Resource 1 includes additional details on the use of Bayesian estimators).

Data was prepared in Stata 15, and analyses were performed in R 4.2.1. Given the use of de-identified data, the local institutional review board deemed the study not human subjects research and exempt.

## Results

There were 4,658,017 births among Black individuals in the USA between 2011 and 2018 in this dataset. After exclusions, there were 4,072,326 births in the analytic dataset, 15.6% of which were among foreign-born individuals. Foreign-born Black birthing people were, on average, older, less likely to be Medicaid-insured and more likely to be self-pay or uninsured, and more highly educated than their US-born counterparts. Prenatal care adequacy was similar between the groups (Table 1).

The PTB rate in the cohort overall was 14.6%, with significantly lower rates among foreign-born (11.4%) compared to US-born Black people (15.2%; Table 1). In unadjusted analyses, immigrants had 28% lower odds of PTB compared to US-born counterparts (OR 0.72, 95% CI 0.71–0.72; Table 2). After adjusting for birthing parents’ sociodemographic and medical covariates, birth state and year and clustering by county, the adjusted odds ratio was slightly attenuated (aOR 0.77, 95% CI 0.76–0.78). Odd ratios comparing PTB among foreign-born to US-born individuals were similar in all the models that included each of the five chosen county-level proxies of advantage and disadvantage (Table 2).

**Fig. 2:**
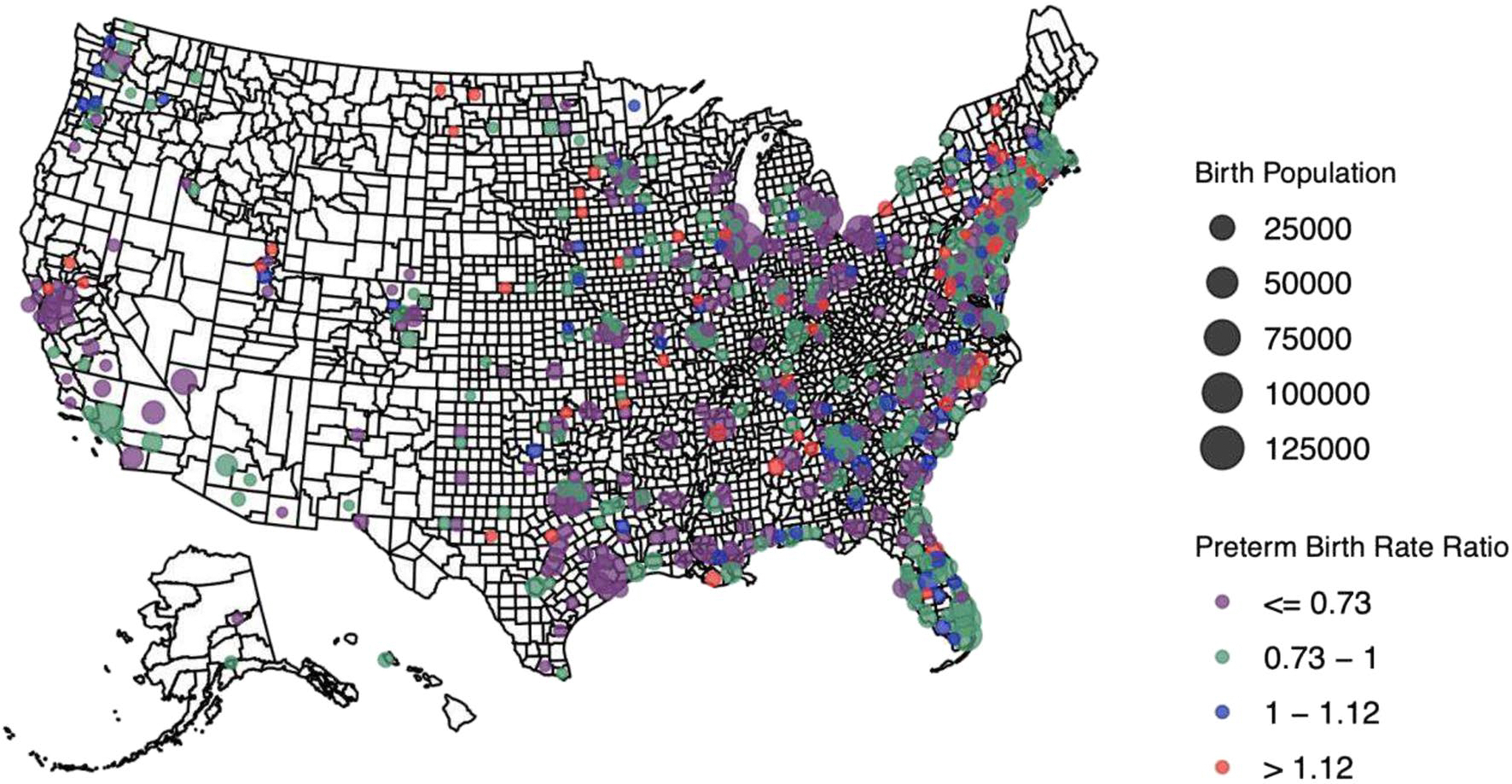
County-level immigrant preterm birth disparity rate ratios across the country. Legend: Dots represents the PTB disparity ratio in each included county. The size of the dot represents the size of the Black birth population. The PURPLE and green dots indicate counties where the rate ratio was < 1 (relative immigrant PTB advantage compared to US-born individuals), while the BLUE and red dots indicate counties where the ratio was > 1 (relative immigrant PTB disadvantage compared to US-born individuals). For more information on the rate ratio, see Online Resource 1 in the Supplement

Of 3143 counties, 622 were included in the exploratory analyses of geographical variation in county-level immigrant PTB advantage. Few counties in the southwest and northwestern regions of the country had enough births to be included (Fig. 2). Among included counties, county disparity rate ratios ranged from 0.13 to 2.82, with 82% demonstrating an immigrant advantage (*n* = 511). The remainder had evidence of a county-level Black immigrant PTB disadvantage (disparity rate ratio was > 1).

County-level percent of Black foreign-born individuals was positively associated with a county-level disparity rate ratio in unadjusted analyses (Table 3, model 11). In other words, a higher proportion of Black foreign-born residents in a county was associated with less of a foreign-born advantage (*β* 0.03, 95% CI 0.10, 0.04) for county-level rates of preterm birth. When adjusting for all county-level predictors together (Table 3, model 13), the log-transformed RR was significantly lower for county-level percent of the population with more than high school education (*β* − 0.06, 95% CI − 0.15, − 0.03). This indicated that this variable was associated with more foreign-born PTB advantage. Conversely, a higher proportion of any foreign-born residents was associated with less of a foreign-born advantage (*β* 0.03, 95% CI 0.01, 0.05) (Table 3, model 13). Sensitivity analyses with Bayesian estimated PTB rate ratios yielded similar results (Online Resource 2).

## Discussion

In this national, multiyear, population-based study, we found that Black immigrant birthing people overall have a lower PTB risk than their US-born counterparts, confirming findings from previous state-based [28, 54] or single-year national cohorts [2]. We further found that Black birthing people’s immigrant PTB advantage compared to US-born individuals was minimally attenuated by adjusting for birthing parents’ sociodemographic and medical characteristics. In smaller studies of Black birthing people that have combined birth certificates with hospital records to obtain birthing parents’ medical data, the nativity advantage has also been mildly attenuated by medical covariates [28]. This mirrors evidence among immigrant Hispanic birthing people [2]. Such findings indicate that drivers of health or disease that exist at the level of the individual alone are insufficient to explain the immigrant paradox. Policies facilitating harmonization between birth certificates and birthing parents’ hospital records at the national level would allow continued exploration of the birthing parents’ medical contribution to the immigrant birth paradox in large datasets.

We also found that higher county-level rates of Black foreign-born individuals were associated with less of a foreign-born advantage, which was unexpected, since this variable was chosen as a proxy for the presumed structural resilience that a racially-concordant immigrant enclave might represent. It is possible that this variable is a poor proxy for the protective nature of a co-ethnic enclave, since it reflects numbers of Black immigrants from any country and region of the world. In contrast to our hypothesis, this county-level variable may reflect structural segregation that still may be useful to immigrant health researchers.

The need to investigate specific immigrant community’s health outcomes within the context of their local community is further supported by our findings that the Black immigrant PTB advantage was not uniform across US counties. Although Black birthing people had lower risk of PTB compared to their US-born counterparts in most counties, there were counties where the PTB rates among foreign-born and US-born individuals were either similar or higher among immigrants. This suggests that the strength of the immigrant paradox (i.e., the protective effect of nativity on adverse outcomes like PTB) may be related to the geographic context in which immigrants are settling in the USA. One possible reason for this may be related to immigrants’ country of origin and the healthy migrant effect, which posits that people who leave their country of birth are healthier than the people who do not emigrate [5]. If specific co-ethnic enclaves can be discerned within counties, then county-level Black immigrant PTB risk may reflect characteristics of immigrants from a particular country. For instance, people from certain countries may have had increased access to health-promoting resources such as universal health care or decreased exposure to toxic stressors like the type of racism experienced by US-born Black people. Another hypothesis is that the communities around certain immigrant groups might be more resourced or health-promoting than those surrounding other immigrant communities. Such resources could reflect the specific social determinants of health in each community, such as local health insurance policies or the presence and strength of local safety net health centers [55].

Finally, we found a significant association between county-level immigrant PTB advantage and increasing county-level rates of individuals with more than high school education. One hypothesis might be that higher numbers of educated individuals in a county may be serving, for instance, as a proxy for the existence of or access to neighborhood resources that are more health-promoting for immigrants than US-born Black individuals and thus creating an unequal area-level, structural resilience.

Our findings also extend the literature around area-level segregation and its association with PTB. We did not find an association between county-level ICE_race_ and county-level immigrant PTB advantage. Similarly, county-level ICE_race_ did not attenuate the odds of PTB among foreign-born Black birthing people compared to US-born counterparts in the individual-level analyses. Previous studies which found associations between ICE_race_ and PTB calculated this proxy for structural racism at the census tract level [47, 56, 57] or within ZIP codes [58] dataset. Although others have found Black-White health disparities related to ICE_race_ at the county-level [59, 60], ICE_race_ may be less useful for the study of disparities within racial groups and/or county-level birth outcomes.

This study has limitations. First, we were restricted to using county-level data to measure structural drivers of risk and resilience due to the limitations of the national birth certificate datasets, but counties across the USA are heterogeneous by size. Second, only 20% of US counties had enough births to Black individuals overall and Black immigrants to be included; however, our cohort represents about 80% of births to Black people reported by the Centers for Disease Control during the study period. Third, though we used multiple cross-sectional measures of structural risk and resilience, these do not take into consideration the cumulative life course exposure to and/or intersectional effects of different forms of discrimination related to gender identity, disability, etc. [17]. There are many other markers of structural discrimination and resilience that can be considered in future studies. Finally, we did not disaggregate the foreign-born subgroup into distinct countries of origin in order to preserve statistical power to detect PTB differences. Thus, our study cannot comment on differential patterns of structural risk and resilience that may exist within immigrants by world region or country of origin. Nonetheless, our work represents one of the only studies with a large enough sample size to explore both individual and structural drivers of PTB among Black birthing people [6]. It was also novel with respect to examining national variation in county-level Black foreign-born PTB advantage and considering area-level factors as potentially representing either structural risk or structural resilience.

## Conclusion

Although it is well established that Black immigrants have a PTB advantage compared to US-born Black individuals, our study provides evidence that the strength of the Black PTB immigrant advantage may be more context-dependent than previously appreciated. Local policies enacted by health departments, city councils, and large healthcare systems may affect Black immigrant birth outcomes and merit exploration in future studies.

## Supplementary Material

Supplementary Material

## Figures and Tables

**Table 1:** Birthing parent characteristics among non-Hispanic Black people with live singleton births from 2011 to 2018 in the USA^a^ (*N* = 4,072,326)

Individual level variables	Overall *N* (%)	US born *N* = 3,438,443 (84.4)	Foreign-born *N* = 633,883 (15.6)	Standardized mean difference^b^

Birthing parent age (years)				0.84
< 20	386,881 (9.5)	376,111 (10.9)	10,770 (1.7)	
20–24	1,195,830 (29.4)	1,125,694 (32.7)	70,136 (11.1)	
25–29	1,135,088 (27.9)	967,800 (28.1)	167,288 (26.4)	
30–34	825,586 (20.3)	616,167 (17.9)	209,419 (33.0)	
≥ 35	528,941 (13.0)	352,671 (10.3)	176,270 (27.8)	
Insurance				0.41
Medicaid	2,662,552 (65.4)	2,340,095 (68.1)	322,457 (50.9)	
Private	1,079,571 (26.5)	875,060 (25.4)	204,511 (32.3)	
Self-pay or other	278,128 (6.8)	184,763 (5.4)	93,365 (14.7)	
Education				0.46
Less than high school	654,170 (16.1)	561,105 (16.3)	93,065 (14.7)	
High school	2,437,256 (59.8)	2,158,701 (62.8)	278,555 (43.9)	
Some college	704,135 (17.3)	519,427 (15.1)	184,708 (29.1)	
Professional degree	216,696 (5.3)	156,318 (4.5)	60,378 (9.5)	
BMI (kg/m^2^)				0.28
< 25.0	1,471,097 (36.1)	1,217,941 (35.4)	253,156 (39.9)	
25.0–29.9	1,038,265 (25.5)	842,175 (24.5)	196,090 (30.9)	
≥ 30.0	1,349,288 (33.1)	1,205,201 (35.1)	144,087 (22.7)	
Smoked during pregnancy	248,594 (6.1)	245,493 (7.1)	3101 (0.5)	0.38
Prenatal care adequacy^c^				0.12
Inadequate	911,258 (22.4)	748,234 (21.8)	163,024 (25.7)	
Moderate	432,624 (10.6)	364,806 (10.6)	67,818 (10.7)	
Adequate	1,222,102 (30.0)	1,029,256 (29.9)	192,846 (30.4)	
Adequate plus	1,228,697 (30.2)	1,062,356 (30.9)	166,341 (26.2)	
Preterm birth	595,571 (14.6)	523,442 (15.2)	72,129 (11.4)	0.11

a Excluding multiples and those with a documented gestational age < 22 weeks or > 45 weeks

b Standardized mean differences > 0.1 can be used to identify covariates that differ between groups in large datasets where *p*-values are very small. All *p*-values were < 0.001

c Calculated using the Adequacy of Prenatal Care Utilization Index which integrates data from the birth certificate about when prenatal care began and the number of prenatal visits

**Table 2:** Odds of preterm birth among foreign-born compared to US-born non-Hispanic Black people (*N* = 4,072,326 people)

Model	Odds ratios (OR) and 95% confidence intervals^b^

Model 1: Unadjusted	0.72 (0.71, 0.72)
Model 2: Adjusted for birthing parent characteristics,^a^ birth year, birth state, congenital anomalies, and clustered by county as a random effect	0.77 (0.76, 0.78)
Model 3: model 2 + county-level percent of people living in poverty	0.77 (0.76, 0.78)
Model 4: model 2 + county-level percent of people without health insurance	0.77 (0.76, 0.78)
Model 5: model 2 + county-level percent of people with more than a high school education	0.77 (0.76, 0.78)
Model 6: model 2 + county-level percent Black foreign born people	0.77 (0.76, 0.78)
Model 7: model 2 + county-level racial polarization (ICE_race_)	0.77 (0.76, 0.78)

a Birthing parent characteristics included age, insurance, education, marital status, pre-pregnancy body mass index, smoking history, and prenatal care adequacy

b All *p*-values were < 0.001

**Table 3:** County-level variables associated with county-level immigrant preterm birth advantage (*n* = 622 counties)

County-level risk and resilience factors^a^		
	*β* (95% CI) of log-transformed rate ratio^b^	P-value

Part A. Unadjusted models with each factor assessed individually		
Model 8: percent living in poverty	− 0.03 (− 0.07, 0.01)	0.10
Model 9: percent without health insurance	− 0.03 (− 0.07, 0.002)	0.06
Model 10: percent with more than high school education	− 0.01 (− 0.05, 0.02)	0.48
Model 11: percent Black foreign-born	0.03 (0.010, 0.04)	0.002
Model 12: racial polarization (ICE_race_)^c^	0.02 (− 0.02, 0.05)	0.29
Part B. Model 13: adjusted model with all factors assessed together		
Percent living in poverty	− 0.03 (− 0.07, 0.02)	0.32
Percent without health insurance	− 0.04 (− 0.09, 0.005)	0.08
Percent with more than high school education	− 0.06 (− 0.10, − 0.01)	0.01
Percent Black foreign-born	0.03 (0.01, 0.05)	< 0.001
Racial polarization (ICE_race_)	0.02 (− 0.03, 0.07)	0.40

a Percentages were standard deviation normalized. Thus, the output represents the change in the PTB rate ratio per standard deviation percent increase in the area level factor

b The immigrant PTB disparity rate ratio is calculated as follows: (Number of PTBs in foreign-born individuals/Number of foreign-born individuals)/(Number of PTBs in US Individuals/Number of US individuals). A rate ratio < 1 indicates that the PTB rate in foreign-born individuals is lower relative to US born individuals and thus that the county has higher immigrant advantage. Conversely, a ratio > 1 indicates a PTB rate in foreign-born individuals higher than among US-born individuals, meaning that county has a lower relative immigrant advantage

c ICE_race_ measures racial polarization in an area by considering both advantage and disadvantage in its calculation. — 1 represents a county where 100% of residents self-reported being Black, and 1 represents a county where 100% of residents self-reported as White

## Data Availability

This study examined restricted use data from the National Vital Statistics Office and thus data must be requested from them through a Data Use Agreement. For more information about analytic code and data definitions, please email the corresponding author.
