# Correction: National Variation in Black Immigrant Preterm Births and the Role of County-Level Social Factors

**DOI:** 10.1007/s40615-024-02222-7

**Published:** 2024-10-22

**Authors:** Ozi Amuzie, Joshua Radack, Nancy Yang, Alejandra Barreto, Daria Murosko, Sara C. Handley, Scott A. Lorch, Heather H. Burris, Diana Montoya-Williams

**Affiliations:** 1 Division of Neonatology, Children’s Hospital of Philadelphia, 2716 South Street, Philadelphia, PA 19146 USA; 2 School of Medicine, University of California, San Francisco, San Francisco, CA USA; 3 Children’s Hospital of Philadelphia PolicyLab, Philadelphia, PA USA; 4 Department of Pediatrics, Perelman School of Medicine at University of Pennsylvania, Philadelphia, PA USA

**Correction: Journal of Racial and Ethnic Health Disparities**

10.1007/s40615-024-02198-4

The surname of author Diana Montoya-Williams was given incorrectly (as “Williams”) in the article as originally published.

The original article has been corrected.

