# Supplementary material for: National Variation in Black Immigrant Preterm Births and the Role of County-Level Social Factors
Source: J Racial Ethn Health Disparities. 2024 Oct 8;12(6):4033–42. doi: 10.1007/s40615-024-02198-4 (PMC11975723; doi:10.1007/s40615-024-02198-4)
Supplement: Supplementary file 1 — Supplementary file1 (DOCX 19 KB) [file 40615_2024_2198_MOESM1_ESM.docx]

**Online Resource: Methodological Considerations**

**The Index of the Concentration of the Extremes (ICE)**

ICE is calculated by the following formula:

ICE_i_ = $\frac{(Ai - Pi)}{Ti}$

*A_i_* represents the number of people or households in the most privileged extreme, *P_i_* represents the number of people or households in the most deprived extreme, and *T_i_* represents the total number of people or households.[^1^](https://paperpile.com/c/dSia9J/Dtdnv) ICE_race_, ICE _income_ , and ICE_race-income_ use Non-Hispanic White persons, households with income >125,000, and Non-Hispanic White households with income >125,000 as the most privileged extreme, respectively, and Non-Hispanic Black persons, households with income <20,000, and Black households with income <20,000 as the most deprived extreme, respectively. Demographic data used to calculate the ICE measures were obtained from publicly available 5-year American Community Survey (ACS) data and matched to each birth by year and county. ICE values were divided by the standard deviation for each measure using data from across the country to obtain standard deviation increments for the exposures for analyses.

**Disparity Rate Ratio**

Disparity rate ratios have been used to compare rates between two cohorts and determine disparities that may exist between these. They have been employed both to determine disparities of a marginalized group relative to a White reference group,[^2^](https://paperpile.com/c/dSia9J/oNJm) but also to elucidate disparities within a certain group by different characteristics such as rurality[^3^](https://paperpile.com/c/dSia9J/JQ9N) or socioeconomic status.[^4^](https://paperpile.com/c/dSia9J/Ue4I) As such it has emerged as a tool to measure inequality or disparity in an outcome.

For our county-level analyses, we defined our outcome variable as the ratio of preterm birth rates between the immigrant and US-born black populations within each county:

$$(\# of Foreign-born PTB/\# of Foreign-born Births)$$

We see that if this county-level disparity rate ratio is less than one, then the numerator (foreign-born PTB rate) is less than the denominator (US-born PTB rate. This indicates a relative county-level immigrant advantage. On the other hand, if the ratio is greater than one, then our PTB disparity rate ratio indicates a relative county-level immigrant disadvantage, as the number of PTBs among foreign-born people in the county is greater relative to the number of PTBs among US-born Black individuals.

**Bayesian Estimators**

Since we restricted to counties with at least 100 births overall and 25 foreign-born births, both denominators of the preterm birth rates are non-zero. However, it is possible for either the number of foreign-born preterm births or the number of US-born preterm births to be zero. The latter situation is particularly problematic since the rate ratio would be undefined due to division by zero. There were five counties that had either zero foreign-born preterm births or zero US-born preterm births.

In order to be able to conduct our analyses without excluding these five counties where there were either zero preterm births among foreign-born or US-born Black individuals, we implemented a Bayesian estimator for the preterm birth rates of both populations. A Bayes estimator combines observed data with prior information to form a posterior estimate of a parameter and can be particularly useful in situations with small sample sizes or rare events.[^5^](https://paperpile.com/c/dSia9J/KjQl) Assuming the data generating process is a binomial distribution for the preterm births, a classic choice for the prior distribution on the probability of a preterm birth would be the beta distribution. To specify the prior hyperparameters, we employed an empirical Bayes approach with prior moment matching method.[^6^](https://paperpile.com/c/dSia9J/8rN6) Using empirical Bayes estimators for the preterm birth rates allows for a simple and intuitive method to correct for the small sample sizes and relatively rare outcomes of Black preterm births within a county. Formulas and derivations to achieve Bayesian estimators for preterm birth rates are available upon request.

**References**

1. [Krieger N, Waterman PD, Batra N, Murphy JS, Dooley DP, Shah SN. Measures of Local Segregation for Monitoring Health Inequities by Local Health Departments. *Am J Public Health*. 2017;107(6):903-906.](http://paperpile.com/b/dSia9J/Dtdnv)

2. [Karaye IM, Maleki N, Yunusa I. Racial and Ethnic Disparities in Alcohol-Attributed Deaths in the United States, 1999-2020. *Int J Environ Res Public Health*. 2023;20(8). doi:](http://paperpile.com/b/dSia9J/oNJm)[10.3390/ijerph20085587](http://dx.doi.org/10.3390/ijerph20085587)

3. [Martens P, Brownell M, Au W, et al. *Health Inequities in Manitoba: Is the Socioeconomic Gap in Health Widening or Narrowing over Time?* University of Manitoba Centre for Health Policy; 2010.](http://paperpile.com/b/dSia9J/JQ9N) <http://mchp-appserv.cpe.umanitoba.ca/reference/Health_Ineq_final_WEB.pdf>

4. [Iqbal MH. Disparities of health service for the poor in the coastal area: does Universal health coverage reduce disparities? *J Mark Access Health Policy*. 2019;7(1):1575683.](http://paperpile.com/b/dSia9J/Ue4I)

5. [Gribok A, Agarwal V, Yadav V. Performance of empirical Bayes estimation techniques used in probabilistic risk assessment. *Reliab Eng Syst Saf*. 2020;201:106805.](http://paperpile.com/b/dSia9J/KjQl)

6. [Siu NO, Kelly DL. Bayesian parameter estimation in probabilistic risk assessment. *Reliab Eng Syst Saf*. 1998;62(1):89-116.](http://paperpile.com/b/dSia9J/8rN6)

| **Online Resource 2. Sensitivity Analyses of County-Level Relationship using Bayesian Estimated PTB Disparity Rate Ratios (n=622 counties)** | |
| --- | --- |
| **County-Level Risk & Resilience Factors^1^** | **County-level Immigrant preterm birth (PTB) Disparity Rate Ratio^2^** |
|  | *Effect estimate (95% Confidence Intervals)* |
| **Part A. Unadjusted models with each factor assessed individually** | |
| Model 8: Percent living in poverty | **-0.03 (-0.05, -0.02)** |
| Model 9: Percent without health insurance | **-0.02 (-0.04, - 0.01)** |
| Model 10: Percent with more than high school education | -0.01 (-0.03, 0.01) |
| Model 11: Percent Black foreign-born | **0.02 (0.01, 0.03)** |
| Model 12: Racial polarization (ICE_Race_)^3^ | **0.02 (0.004, 0.04)** |
| **Part B. Model 13: Adjusted model with all factors assessed together** | |
| Percent poverty | **-0.03 (-0.06, -0.01)** |
| Percent without health insurance | -0.02 (-0.04, 0.006) |
| Percent with more than high school education | **-0.04 (-0.06, -0.02)** |
| Percent Black foreign-born | **0.02 (0.01, 0.03)** |
| Racial polarization (ICE _Race_) | 0.01 (-0.001, 0.05) |

^1^ Percentages were standard deviation normalized. Thus, the output represents the change in the PTB rate ratio per standard deviation percent increase in the area level factor.

^2^The Immigrant PTB Disparity Rate Ratio is calculated as: (Number of PTBs in foreign-born individuals/Number of foreign-born individuals)/(Number of PTBs in US Individuals/Number of US individuals). A rate ratio < than 1 indicates that the PTB rate in foreign born individuals is lower relative to US born individuals and thus that the county has higher immigrant advantage. Conversely, a ratio > 1 indicates a PTB rate in foreign-born individuals higher than among US-born individuals, thus making that county have lower immigrant advantage.
